# Born to Yawn? Understanding Yawning as a Warning of the Rise in Cortisol Levels: Randomized Trial

**DOI:** 10.2196/ijmr.2241

**Published:** 2012-09-20

**Authors:** Simon BN Thompson, Phil Bishop

**Affiliations:** 1 Psychology Research Centre& Dementia InstituteBournemouth UniversityPooleUnited Kingdom; 2 Psychology Research CentreBournemouth UniversityPooleUnited Kingdom

**Keywords:** Clinical practice, cortisol, electromyography, neural pathway, saliva, yawning

## Abstract

**Background:**

Yawning consistently poses a conundrum to the medical profession and neuroscientists. Despite neurological evidence such as parakinesia brachialis oscitans in stroke patients and thermo-irregulation in multiple sclerosis patients, there is considerable debate over the reasons for yawning with the mechanisms and hormonal pathways still not fully understood. Cortisol is implicated during yawning and may link many neurological disorders. Evidence was found in support of the Thompson cortisol hypothesis that proposes cortisol levels are elevated during yawning just as they tend to rise during stress and fatigue.

**Objectives:**

To investigate whether saliva cortisol levels rise during yawning and, therefore, support the Thompson cortisol hypothesis.

**Methods:**

We exposed 20 male and female volunteers aged between 18 and 53 years to conditions that provoked a yawning response in a randomized controlled trial. Saliva samples were collected at the start and again after the yawning response, or at the end of the stimuli presentations if the participant did not yawn. In addition, we collected electromyographic data of the jaw muscles to determine rest and yawning phases of neural activity. Yawning susceptibility scale, Hospital Anxiety and Depression Scale, General Health Questionnaire, and demographic and health details were also collected from each participant. A comprehensive data set allowed comparison between yawners and nonyawners, as well as between rest and yawning phases. Collecting electromyographic data from the yawning phase is novel, and we hope this will provide new information about neuromuscular activity related to cortisol levels. Exclusion criteria included chronic fatigue, diabetes, fibromyalgia, heart conditions, high blood pressure, hormone replacement therapy, multiple sclerosis, and stroke. We compared data between and within participants.

**Results:**

In the yawning group, there was a significant difference between saliva cortisol samples (*t*_10 _= –3.071, *P *= .01). Power and effect size were computed based on repeated-measures *t *tests for both the yawning and nonyawning groups. There was a medium effect size for the nonyawners group (*r *= .467) but low power (36%). Results were similar for the yawners group: medium effect size (*r *= .440) and low power (33%).

**Conclusions:**

There was significant evidence in support of the Thompson cortisol hypothesis that suggests cortisol levels are elevated during yawning. A further longitudinal study is planned to test neurological patients. We intend to devise a diagnostic tool based on changes in cortisol levels that may assist in the early diagnosis of neurological disorders based on the data collected.

**Trial Registration:**

International Standard Randomized Controlled Trial Number (ISRCTN): 61942768; http://www.controlled-trials.com/ISRCTN61942768/61942768 (Archived by WebCite at http://www.webcitation.org/6A75ZNYvr)

## Introduction

Yawning consistently poses a conundrum to neurologists and neuroscientists [1]. Increasingly, evidence is found to link neurological disorders through the commonality of yawning episodes and contagious yawning. Despite discrete incidences (such as parakinesia brachialis oscitans) in brain stem ischemic stroke patients, there is considerable debate over the reasons for yawning, with the mechanism of yawning still not fully understood [2]. Cortisol is implicated during yawning and may link many neurological disorders. Evidence was found in support of the Thompson cortisol hypothesis [3,4] that proposes cortisol levels are elevated during yawning just as cortisol levels are known to be raised in instances of stress and fatigue [5].

There have been several explanations about the yawning mechanism. Yawning is a physiological behavior that has been described as a transition between wakefulness and sleep [6]. According to Walusinski [7], yawns exteriorize the activity of the motor centers of the brain stem (cranial nerves V, VII, IX, X, XI, and XII) and of the spinal cord under the control of the hypothalamic paraventricular nucleus. The hypothalamic paraventricular nucleus is a point of integration between the central and peripheral autonomic systems. Walusinski [7] comprehensively presents several disorders due to deregulation of yawning:

- anhedonia (frustration because of an incomplete or inharmonious development of a yawn possibly due to unconscious inhibition of the letting go that underlies a complete yawn)
- the disappearance of a yawn (indicating the activity state of the dopaminergic neurons of the hypothalamic paraventricular nucleus, which are necessary for yawning)
- excessive yawning (possibly linked to hunger and arousal) and famously illustrated in Charcot’s *Leçons du Mardi de la Salpêtrière* [8] by his patient who yawned eight times in a minute.

Yawning is a powerful reflex that may serve to evacuate the palatine tonsillar fossae [9]. This is a possible explanation because the strong reflex does not have any immediate urgency, is reflected in our circadian rhythm, and is allocated to times that cause us minimal inconvenience. However, McKenzie [9] suggests that, due to our social sanctions in the Western world to generally suppress the yawning reflex, perhaps we are leading to endemic tonsillitis.

Spontaneous yawning is present in humans from the early stages of development [10]. It has been observed in infants and newborns and in fetuses of 12- to 14-weeks’ gestational age. The time course of yawning seems to differ with age [6], with adults yawning in the early morning and late evening [11]; in young adults, yawning seems to be linked to a low level of vigilance, increasing before and after the sleep episode [12]. Yawning is also contagious and can be elicited by seeing or even hearing someone else yawn [13]. Yet yawning has also been observed in other species [14,15], which has led to suggestions that yawning may serve communicative as well as physiological functions.

Some authors do believe that the physiological explanations given by some researchers do not adequately explain the reasons why we yawn. For example, Guggisberg and colleagues [16] argue that research tends to support yawning as a communicative function. Gallup [17] suggests it is likely that there is not just one theory to explain the functions of yawning, and it is unlikely that yawning serves primarily as a communicative function, since experimental evidence of contagious yawning is observed in only a small number of species in one lineage (primates). We tend to agree with this notion and believe the evidence of yawning in patients with neurological disorders and stroke (such as parakinesia brachialis oscitans) tends to suggest that there are specific mechanisms for yawning that are excited under special circumstances. However, it is acknowledged that yawning may be elicited because of empathy in *Homo sapiens *[18,19].

The debate for clarity continues [20]. Some authors argue that physiological explanations are imprecise and that there is evidence that neural networks responsible for empathy and social skills may be implicated and activated during the yawning episode. There is a problem with comparing some studies due to methodological differences and inadequacies [21].

However, evidence from neurological patients has led to a new line of enquiry that focuses on thermoregulation. Corey and colleagues [22] examined physiological measurements taken before, during, and after yawns in humans. They concluded their data are most consistent with the brain-cooling hypothesis and advocate that yawning increases blood flow. Indeed, it is known that painful headaches [23] and thermoregulatory disorders [24] may arise from excessive yawning. Corey and colleagues [22] suggest that the yawning experienced during these times may be due to circulatory dysfunction.

Gallup and Gallup [25] reported on repetitive yawning in patients with multiple sclerosis, showing that thermoregulatory dysfunction is a symptom of multiple sclerosis. Furthermore, yawning seems to provide symptom relief in patients with multiple sclerosis. Gallup and Eldakar [26] also showed that the incidence of yawning in humans is associated with seasonal climate variation.

Researchers are constantly striving to find commonality in disorders via their metabolic or neuronal pathways. It is interesting to note in past years how the treatment of Parkinson disease could be modified because of the exploration of dopaminergic and serotonic pathways [1,27,28]. Uncertainty in the functions of some neurotransmitters and their possible multiple implications in chemical pathways presents a complicated picture that is not unlike the clinical signature of Alzheimer disease in those with comorbid Down syndrome [29]. Having Alzheimer disease together with Down syndrome does not necessarily result in the clinical symptoms of dementia in later life [30].

The overlap between symptoms and neurochemical pathways may be more apparent than initially thought. New evidence has emerged of overlapping pathways involving DISC1, a scaffold protein that interacts with multiple neurodevelopmental, cytoskeletal, and signaling proteins [31], and Huntingdon disease [32]. In time, it is hoped that yawning and its role in neurological disorders may be understood by exploring its presence as a symptom in different neurological disorders.

Indeed, Collins and Eguibar [33] stated that antagonist interaction studies have now clearly defined at least 3 distinct neural pathways involved in the induction of yawning. Scientists are beginning to understand the hierarchical order through which these different neurotransmitter systems interact to regulate yawning. So far, the following neurotransmitters and neurohormones have been implicated: acetylcholine, dopamine, glutamate, serotonin, oxytocin, gamma-aminobutyric acid, opioids, adrenergics, nitric oxide, adrenocorticotropic hormone, and alpha-melanocyte stimulating hormone. Yet advanced techniques, such as functional magnetic resonance imaging, are yet to yield conclusive evidence to assist in fully explaining the yawn [34].

Yawning has often been associated with fatigue, stress, and exposure to cold [1]. During exposure to cold, the cortisol level in humans rises dramatically, except when they are exposed quickly (as in the cold face test), when there are reduced cortisol rises, perhaps due to vagal inhibition [35]. It is suspected that exposure to extreme cold temperature gives rise to a similar stress-like response with respect to cortisol levels in humans.

Cortisol is known to be present and elevated during stressful situations. Blood cortisol levels are directly related to salivary cortisol levels [36], which have been documented in various paradigms. The cortisol level and stress correlation is curvilinear. However, in preterm infants, cortisol levels may be lower during the heel-stick pain procedure [37], and in girls whose parents had depressive problems, cortisol levels were blunted [38]. In animal models, the cortisol level profile is also similar to that in humans during stressful situations [39]. Cortisol levels appear higher after being subjected to stress-induced situations [40].

What is unknown is the cortisol level during yawning. The cortisol level may be higher when yawning occurs after exposure to cold than after exposure to a stressful situation. Are cortisol levels elevated when neurologically impaired patients yawn, perhaps in those with multiple sclerosis?

The link between fatigue and hormonal changes is well documented. A greater level of neuromuscular fatigue and larger responses in serum hormone concentrations have been seen, for example, after hypertrophic variable resistance loadings [41]. This has led to identifying markers of fatigue [42], particularly following postmatch professional rugby [43] and in young athletes [44]. Elevated salivary cortisol levels have also been seen in elite tennis players [45]. Sleep deprivation and fatigue have been linked with salivary cortisol levels; in this instance, cortisol levels are lowered [46].

Cortisol is a lipophilic steroid with low molecular weight. Following binding with adrenocorticotropic hormone to membrane receptors and cells of the adrenal cortex, cortisol is then synthesized and released into the blood stream. Since most (about 95%) is bound to large proteins, such as albumin, only the small fraction of unbound free cortisol is thought to be biologically active *and *enters cells by passive diffusion. This makes it feasible to measure the free cortisol fraction in all bodily fluids, for example, saliva [47].

Levels of cortisol are regulated by the hypothalamic-pituitary-adrenal axis, which is a complex set of interactions between the hypothalamus (known to regulate body temperature) and the pituitary and adrenal glands. The hypothalamic-pituitary-adrenal axis also assists in digestion, the immune system, sexuality, mood, and energy usage [48]. It is implicated in stress, trauma, and particular disorders such as fibromyalgia and chronic fatigue syndrome.

Curiously, the compound glycyrrhizic acid, found in licorice, has been found to increase the activity of cortisol in the kidney [49]. This is thought to be due to inhibition of the enzyme 11Β-hydroxysteroid dehydrogenase type 2, which normally inactivates cortisol in the kidney; hence, licorice tends to inhibit this enzyme and in turn deregulates, resulting in an increase of, cortisol levels. Anecdotally, we asked 1 of our study participants to eat licorice after providing a saliva sample and observed a rise in cortisol levels. This would need to be investigated further to discern significance in this finding.

## Methods

We recruited 20 male and female volunteers aged between 18 and 53 years from students at Bournemouth University, Poole, UK, using a computerized recruitment system (Sona Systems, Tallinn, Estonia) and Facebook. Consent from all participants was properly obtained according to code of conduct and research guidelines. Participants were exposed, under randomized controlled trials guidelines, to three conditions intended to provoke a yawning response: photos of people yawning; boring text about yawning; and a short video of a person yawning. Comparisons were made with people exposed to the same conditions but who did not yawn.

We collected saliva samples at the start and again after a yawning response, together with electromyographic data of the jaw muscles via surface-placed electrodes to determine rest and yawning phases of neural activity. If there was no yawning response, then we took a second saliva sample at the end of the experimental paradigm. A yawning susceptibility scale (questionnaire designed for this study), Hospital Anxiety and Depression Scale (HADS) [48,50], General Health Questionnaire-28 (GHQ-28) [51-56], and demographic and health details were also collected from each participant.

Exclusion criteria were chronic fatigue, diabetes, fibromyalgia, a heart condition, high blood pressure, hormone replacement therapy, multiples sclerosis, and stroke. Between- and within-participant comparisons were made using *t *tests, and correlations were calculated using the SPSS package, version 19 (IBM Corporation, Somers, NY, USA). This enabled a comparison to be made between yawner and nonyawner participants, as well as between rest status and yawning episodes.

### Ethics

Bournemouth University research and ethics approval was granted (BU-PS5/10/11; PS1/3/12). Professional code of conduct, confidentiality, and safety issues were addressed and approved in the ethics submission. Data collected were made anonymous, coded, securely stored, and destroyed after completion of the study analysis. Protective measures were put in place for collection and analysis of saliva samples, and the right of participants to withdraw from the study was made clear to all participants.

### Funding

This research received funding of £4000 from the host institution, Bournemouth University to support the purchase of essential equipment and materials. In addition, £4344 was received from Santander plc for travel expenses incurred to assist the first author to gain essential information for the selection and analysis of salivary cortisol kits.

## Results

In saliva cortisol sample 1, the mean for nonyawners (n=9) was 3.3889 (SD 1.43479) and for yawners (n=11) was 2.9727 (SD 1.94889). In sample 2, the means were 4.5778 (SD 1.93589) for nonyawners and 3.9273 (SD 2.36309) for the yawners (Table 1).

There were no significant differences between groups in terms of age (*t*_18 _= –0.071, *P *= .94), HADS depression scores (*t*_18 _= 0.890, *P *= .39), GHQ-28 scores (*t*_18 _= 0.663, *P *= .52), or HADS anxiety scores (*t*_18 _= 0.484, *P *= .63). Age, depression, GHQ-28 total, and anxiety scores were not significantly correlated with sample 1 (saliva cortisol) (Table 2).

**Table 1:** Descriptive statistics of participants.

Yawn		Mean	SD	n
**Sample 1**				
	Nonyawn	3.3889	1.43479	9
	Yawn	2.9727	1.94889	11
	Total	3.1600	1.70615	20
**Sample 2**				
	Nonyawn	4.5778	2.93589	9
	Yawn	3.9273	2.36309	11
	Total	4.2200	2.58428	20

**Table 2:** Correlations between independent variables (n = 20) for sample 1.

Variable		Age	General total	Anxiety	Depression	Sample 1	
**Age**							
	Pearson correlation	1	–.428	–.709^a^	–.582^a^	.093
	*P *value (2-tailed)		.06	.0	.01	.70
**General total**							
	Pearson correlation	–.428	1	.425	.790^a^	–.185
	*P *value (2-tailed)	.06		.06	.0	.44
**Anxiety**							
	Pearson correlation	–.709^a^	.425	1	.750^a^	–.011
	*P *value (2-tailed)	.0	.06		.0	.96
**Depression**							
	Pearson correlation	–.582^a^	.790^a^	.750^a^	1	–.005
	*P *value (2-tailed)	.01	.0	.0		.98
**Sample 1**							
	Pearson correlation	.093	–.185	–.011	–.005	1
	*P *value (2-tailed)	.70	.44	.98	.98	

^a ^Correlation is significant at the 0.01 level (2-tailed).

Results were normally distributed, as illustrated by the Shapiro-Wilk analysis (Table 3). Results for sample 1 (saliva cortisol taken at the start) suggest that there were no significant differences between those who yawned and those who did not, as confirmed by an independent-samples *t *test (*t*_18 _= 0.532, *P *= .60). An independent-samples *t *test suggests that there were no significant differences in sample 2 (saliva cortisol taken either after yawning or after the last stimuli presentation if the participant did not yawn) between those who yawned and those who did not (*t*_18 _= 0.550, *P *= .59).

**Table 3:** Normality testing of data.

Sample		Kolmogorov-Smirnov^a^			Shapiro-Wilk		
		Statistic	*df*	*P *value	Statistic	*df*	*P *value	
**Sample 1**							
	Nonyawn	.199	9	.20^b^	.901	9	.28
	Yawn	.218	11	.15	.919	11	.31
**Sample 2**							
	Nonyawn	.234	9	.17	.836	9	.05
	Yawn	.152	11	.20^b^	.939	11	.51

^a ^Lilliefors significance correction.

^b ^This is a lower bound of the true significance.

There were no significant differences in saliva cortisol between sample 1 and sample 2 for those who did not yawn during the experiment. This was confirmed using a repeated-measures *t *test (*t*_8 _= –1.710, *P *= .13; Table 4).

**Table 4:** Paired-samples test for nonyawners and yawners (sample 1 – sample 2).

Study group	Mean	SD	SEM	95% CI^a ^of the difference	*t *Test	*df*	*P *value (2-tailed)
Nonyawners	–1.18889	2.08533	.99511	–2.79182 to .41404	–1.710	8	.13
Yawners	–.95455	1.03089	.31082	–1.64710 to –.26199	–3.071	10	.01

^a ^Confidence interval.

However, there was a significant difference in saliva cortisol between sample 1 and sample 2 among the yawners (*t*_10 _= –3.071, *P *= .01; Table 4).

### Power and Effect Size

We computed power and effect size based on repeated-measures *t *tests for both the yawning and nonyawning groups. There was a medium effect size for the nonyawners (*r *= .467) but low power (36%). Results were similar for the yawners: medium effect size (*r *= .440) and low power (33%).

### Saliva Cortisol Samples

Correlations between participants’ HADS anxiety scores and sample 1 (saliva cortisol) were not significant (*r *= –.11, n = 20, *P *= 0.96); nor were correlations between participants’ HADS depression scores and sample 1 (saliva cortisol; *r *= –.005, n = 20, *P *= .98).

Correlations between participants’ GHQ-28 total score and sample 1 (saliva cortisol) were not significant (*r *= –.185, n = 20, *P *= .44); nor were correlations between age and sample 1 (saliva cortisol; *r *= .93, n = 20, *P *= .70).

Therefore, it was unnecessary to run an analysis of covariance, as none of the covariates were sufficiently correlated.

### Normal Distribution Test

Sample 1 (saliva cortisol) was normally distributed across the whole group of participants (W (20) = 0.939, *P *= .23). It was normally distributed for the nonyawners (W (9) = 0.901, *P *= .26) and for the yawners (W (110) = 0.919, *P *= .31).

Sample 2 (saliva cortisol) was normally distributed (W (200) = 0.929, *P *= .15) across the whole group. It was normally distributed for the nonyawners (W (90) = 0.836, *P *= .53) and for the yawners (W (110) = .0939, *P *= .51).

### t Tests: Between Groups

For the yawners versus nonyawners, there was no significant difference between the groups for sample 1 (saliva cortisol; *t*_180 _= 0.532, *P *= .60) or for sample 2 (saliva cortisol; *t*_18 _= 0.550, *P *= .59).

### t Tests: Within Groups

There was no significant difference between sample 1 (saliva cortisol) and sample 2 (saliva cortisol) in the nonyawning group (*t*_8 _= –1.710, *P *= .13). However, in the yawning group, there was a significant difference between samples (*t*_10 _= –3.071, *P *= .01).

## Discussion

Several interesting findings have emerged from the study, which are consistent with the original hypothesis. Among those who yawned, there was a significant difference in cortisol levels between sample 1 and sample 2. A *t *test confirmed that there were no significant differences in salivary cortisol levels between those who yawned and those who did not for the first baseline sample (sample 1). Furthermore, there were no significant differences in a repeated-measures *t *test between sample 1 and sample 2 for those who did not yawn.

It was a concern while designing the study that age, recent anxiety and depression levels, and general health could all potentially be factors affecting participants’ baseline cortisol levels. If this were the case, these factors could also have contributed toward the change in cortisol levels, and the experiment could have been measuring interference from these, rather than a change due to the independent variable (whether or not participants yawned during the experiment). However, correlation analysis suggested that these did not play a significant part in cortisol levels, with nonsignificant correlations across all variables. There were no significant differences in baseline cortisol levels between those who yawned and those who did not.

Age, recent anxiety and depression levels, or general health could also have had a potential impact on cortisol levels, which could have affected whether a participant yawned or not during the study. The *t *tests for each of the above variables confirmed that there were no significant differences in these factors between groups.

Although we used a relatively low sample size, inspection of data (Table 3) suggests that the differences between groups in terms of cortisol change between sample 1 and 2 were not vast. We intend to investigate these findings further by conducting the experiment on a larger scale, with a proposed 100 participants. Calculation using G*Power suggests that for a power size of 80%, 27 participants will be required for each group; therefore, 100 participants should permit random allocation of 50 participants per group. Investigation of participants with different neurological disorders, such as multiple sclerosis, is also planned. We hope to achieve an understanding of yawning and its role in neurological disorders, together with the potential development of a diagnostic test for the early identification of neurological sequelae.

As well as being of interest to clinical scientists and practitioners, yawning is clearly of interest in the media. A newspaper article has cited interest by the US Department of Homeland Security, which warned that apparently innocuous yawning behavior in passengers could signal a would-be terrorist [57]. Although this reported observation can be argued as perhaps being rather tangential to the pursuit of an explanation of yawning, this tends to highlight the importance of other contributing factors such as the social milieu and cultural norms.

Social and physiological factors as well as cortisol activity are all important considerations, not only because they may potentially provide the answer to why we yawn but also because they may help in the development of a potential diagnostic test. The research team led by the first author at Bournemouth University is interested in determining whether we are truly born to yawn as a protective indicator of untoward neurological dysfunction. Yawning is perhaps a warning, neurologically speaking.

## Abbreviations

GHQ-28: General Health Questionnaire-28
HADS: Hospital Anxiety and Depression Scale
